# Novel Radiolytic Rotenone Derivative, Rotenoisin B with Potent Anti-Carcinogenic Activity in Hepatic Cancer Cells

**DOI:** 10.3390/ijms160816806

**Published:** 2015-07-24

**Authors:** Srilatha Badaboina, Hyoung-Woo Bai, Yun Hee Na, Chul-Hong Park, Tae Hoon Kim, Tae-Hoon Lee, Byung Yeoup Chung

**Affiliations:** 1 Advanced Radiation Technology Institute, Korea Atomic Energy Research Institute, Jeongeup 580-185, Korea; E-Mails: srilatha_bio@rediffmail.com (S.B.); hbai@kaeri.re.kr (H.-W.B.); nayoon@kaeri.re.kr (Y.H.N.); parkch@kaeri.re.kr (C.-H.P.); 2 Interdisciplinary Graduate Program in Molecular Medicine, Chonnam National University, Gwangju 501-746, Korea; E-Mail: thlee83@chonnam.ac.kr; 3 School of Biological Sciences and Biotechnology, Chonnam National University, Gwangju 500-757, Korea; 4 Department of Food Science and Biotechnology, Daegu University, Gyeongsan-si, 712-714, Korea; E-Mail: skyey7@daegu.ac.kr; 5 Department of Biochemistry, School of Dentistry, Chonnam National University, Gwangju 500-757, Korea

**Keywords:** rotenone, rotenoisin B, apoptosis, hepatic cancer, Huh7, pAKT, mitogen activated protein kinase (MAPK)

## Abstract

Rotenone, isolated from roots of derris plant, has been shown to possess various biological activities, which lead to attempting to develop a potent drug against several diseases. However, recent studies have demonstrated that rotenone has the potential to induce several adverse effects such as a neurodegenerative disease. Radiolytic transformation of the rotenone with gamma-irradiation created a new product, named rotenoisin B. The present work was designed to investigate the anticancer activity of rotenoisin B with low toxicity and its molecular mechanism in hepatic cancer cells compared to a parent compound, rotenone. Our results showed rotenoisin B inhibited hepatic cancer cells’ proliferation in a dose dependent manner and increased in apoptotic cells. Interestingly, rotenoisin B showed low toxic effects on normal cells compared to rotenone. Mitochondrial transmembrane potential has been decreased, which leads to cytochrome c release. Down regulation of anti-apoptotic Bcl-2 levels as well as the up regulation of proapoptotic Bax levels were observed. The cleaved PARP (poly ADP-ribose polymerase) level increased as well. Moreover, phosphorylation of extracellular signal regulated kinase (ERK) and p38 slightly up regulated and intracellular reactive oxygen species (ROS) increased as well as cell cycle arrest predominantly at the G_2_/M phase observed. These results suggest that rotenoisin B might be a potent anticancer candidate similar to rotenone in hepatic cancer cells with low toxicity to normal cells even at high concentrations compared to rotenone.

## 1. Introduction

Cancer is the continuous process of uncontrolled growth of abnormal cells in any part of the body. Despite advances in novel therapeutic agents, hepatic cancer is the most aggressive malignant tumor worldwide and remains the fifth most deadly cancer, higher in incidence in men than in women by a factor of two to six among various ethnic groups [1]. Chemotherapy plays an important role in the treatment of cancer, but it is limited to a significant extent by its toxicities, significant resistance of cancer cells to available chemotherapeutic agents, and side effects [2,3]. To increase the efficacy of anticancer drugs to decrease toxicities and side effects is to develop traditional medicines, especially from medicinal plants [4].

Rotenone is a polyphenolic compound, extracted from the roots, leaves, and seeds of leguminosae family plants [5], which is generally considered as a botanical insecticide. Although rotenone possess various biological activities, it is toxic to pests and beneficial insects, birds, aquatic animals like fish and some mammals. Its oral lethal dose for rats ranges from 132 to 1500 mg/kg [6]. After prolonged systemic administration, rotenone could induce all the symptoms of Parkinson’s disease as well [7]. Following these observations, in some states of the USA, its use has been limited or temporarily prohibited by United States Environmental Protection Agency. Above all negatives, it has shown great potential as a chemo preventive and therapeutic agent against several types of cancers, including colon, lung, and breast [8,9,10]. Rotenone induces apoptosis in human breast cancer cells mediated by reactive oxygen species (ROS) through c-Jun N-terminal kinase (JNK) and p38 Signaling [10] whereas, in hepatic cancer cells, it encourages inhibition of mitochondrial complex I and could increase intracellular ROS generation [11].

Lots of drug candidates have been developed, however many of them were banned because of their unexpected adverse effect during clinical trials. Recently, several research groups attempted to generate new useful compounds by structural modifications instead of chemical synthesis. However, little attention has been paid to use radiation in order to encourage transformation. It was verified that gamma radiation is a powerful tool to modify compounds, enhancing their bioactivity [12]. Gamma irradiation of rotenone in methanol solution leads to the formation of a new rotenone derivative product, named rotenoisin B, which was isolated by column chromatographic purification. The rotenosin B contains rare functional groups instead of a ketone moiety at the C-12 of rotenone. Rotenoisin B was a yellowish powder with molecular formula C_27_H_28_O_9_ [13]. The present study aimed to investigate the effect of rotenoisin B, radialytic rotenone, on anti-carcinogenic activity and its action mechanisms in hepatic cancer cells with low toxic effect on the normal cells compared to rotenone.

## 2. Results and Discussion

### 2.1. Cytotoxicity Induced by Rotenoisin B Compared with Parent Rotenone

**Figure 1:**
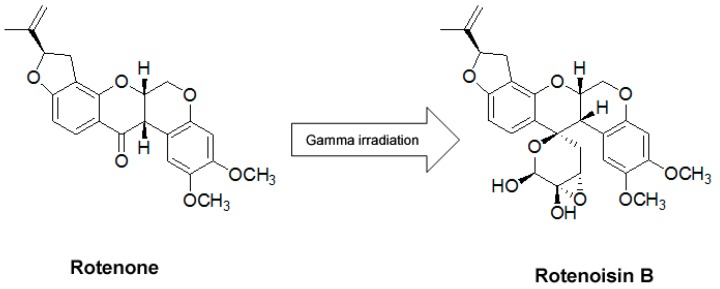
The chemical structures of rotenone and rotenoisin B.

Recently, we demonstrated that radiolytic transformation of the isoflavonoid rotenone with gamma irradiation afforded degraded product, named rotenoisin B. The structure of the new rotenone derivative was elucidated on the basis of spectroscopic methods (Figure 1) [13]. In this study we were interested to find out the anticancer properties of rotenoisin B and the molecular mechanisms by which it mediates apoptosis in hepatic cancer cells.

**Figure 2:**
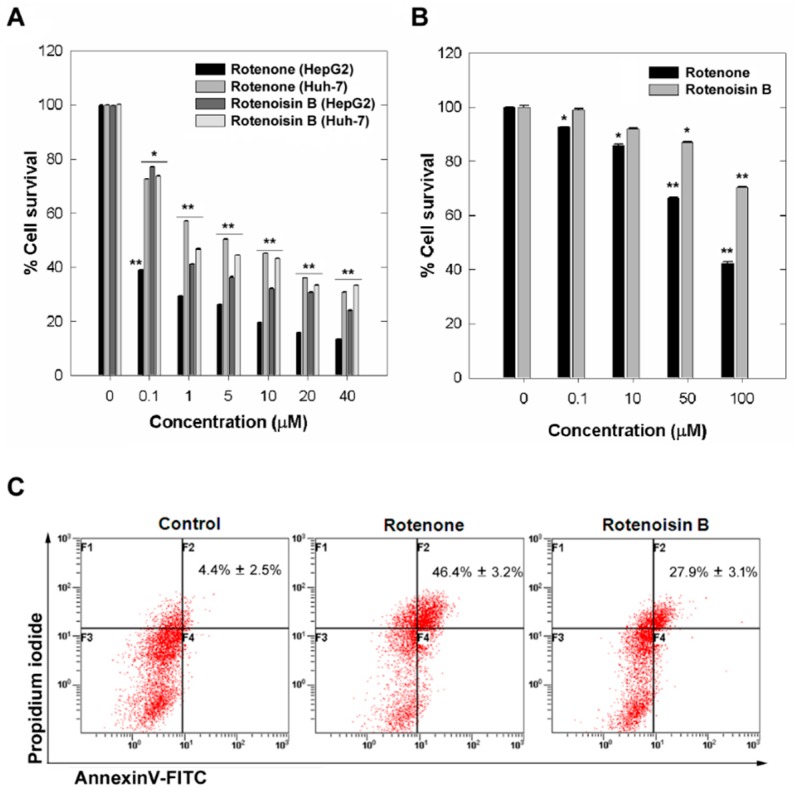
The effect of rotenone and rotenoisin B on the viability of Hepatic cancer cells. (**A**) Cell viability detection by MTT assay in HepG2 and Huh-7 cells after treatment with rotenone and rotenoisin B for 48 h. Each bar indicates means ± SD of four separate experiments. Significant differences from untreated control were indicated by * *p* < 0.05; ** *p* < 0.01; (**B**) Cell viability detection in Detroit 551 cells; and (**C**) The percentage of early and late apoptotic cells detection by double staining with annexin V-FITC and PI after Huh-7 cells treatment with rotenone and rotenoisin B. Bottom right, annexin V-FITC positive only considered as early apoptotic cells; top right, annexin V-FITC/PI double-positive considered late apoptotic cells. The percentage of early and late apoptotic cells combinedly indicated. The results represent as the mean ± S.D. of three independent experiments.

To study the rotenoisin B and rotenone induced proliferation inhibition in hepatic cancer cell lines, HepG2 and Huh-7 cells were exposed to various concentrations of rotenoisin B and rotenone for 48 h. The results from the MTT assay showed that both reduced cell viability in a concentration-dependent manner (Figure 2A). The reduction reached 39.07% ± 0.4% at 0.1 μM rotenone treatment whereas 1 μM rotenoisin B treatment resulted in reduction of viability to 41.16% ± 0.06% in HepG2 cell line compared to DMSO control. In case of Huh-7 cell line, reduction reached 45.11% ± 0.01% at 10 μM rotenone treatment whereas 10 μM rotenoisin B treatment resulted in reduction of viability to 43.31% ± 0.02% compared to control.

Furthermore, rotenone being pesticide having toxicological data showed neurotoxic actions that could play a role in the development of Parkinson disease [14]. We have found that the administration of 50 μM concentration rotenoisin B for 48 h to normal cell line Detroit 551, human fibroblast cells, did not cause cytotoxicity, and even at 100 μM concentration rotenoisin B caused only about 20% cell death, whereas 50 μM higher concentrations rotenone was cytotoxic to normal Detroit 551 cells reduced viability 46.54% ± 0.05% (Figure 2B). We subsequently assessed the anti-cancer effects by annexin V-FITC (fluorescein isothiocyanate) and PI (propidium iodide) with flow cytometry. Our results showed both rotenone and rotenoisin B increased apoptotic cells. Rotenone treatment resulted increase from 4.4% ± 2.5% to 46.4% ± 3.2% whereas as from 4.4% ± 2.5% to 27.9% ± 3.1% upon rotenoisin B treatment of Huh-7 cells for 48 h (Figure 2C).

### 2.2. Effect of Rotenoisin B on Ψ_m_, ROS and Signaling Pathways

**Figure 3:**
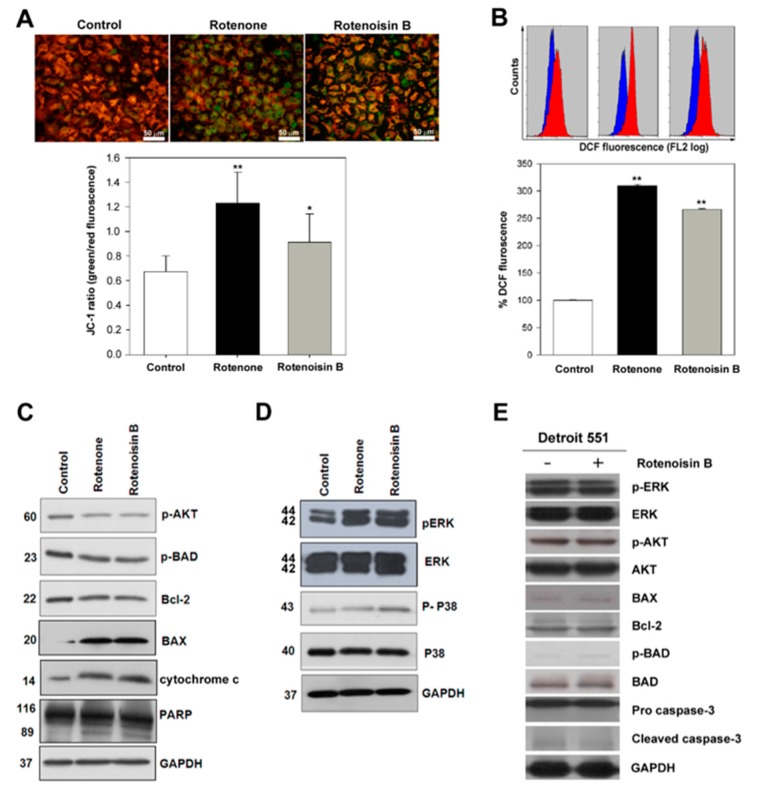
Mitochondrial transmembrane potential of rotenone and rotenoisin B by JC-1dye (**A**) analyzed with inverted fluorescence microscopy. The ratio of green to red fluorescence calculated; (**B**) Intracellular ROS detection using H2DCFDA (2′,7′-dichlorodihydrofluorescein diacetate) by flow cytometry. Values were compared to the control and expressed as the means ± S.D of three independent experiments (* *p* < 0.05, ** *p* < 0.01); (**C**) Immunoblot analysis of p-AKT (Thr 308), AKT, p-BAD (Ser 136), Bcl-2, BAX, cytochrome c and GAPDH; (**D**) Immunoblot analysis of pERK, ERK, p38, p-p38 and GAPDH after Huh-7 cells treatment with rotenone and rotenoisin B; (**E**) Immunoblot analysis of the molecules associated with cell proliferation and apoptosis in Detroit 551 cells after treatment with rotenoisin B.

Many research studies showed that rotenone is an inhibitor of the mitochondrial electron transport chain complex I, resulting in the generation of reactive oxygen species (ROS) [15], and increased intracellular ROS led to apoptotic cell death [16,17]. As showed in Figure 3A, our results indicated decrease in mitochondrial transmembrane potential in Huh-7 cells after rotenoisin B and rotenone treatment for 4 h, stained with JC-1 and then analyzed with inverted florescence microscopy. Green fluroscence increase was significantly observed in rotenone and rotenoisin B treatment compared to the control. To confirm these results, spectroscopy analysis done after JC-1 staining. The ratio of green to red fluorescence was increased from control 0.62 ± 0.12 to 0.91 ± 0.23 in case of rotenoisin B treatment whereas from 0.62 ± 0.12 to 1.23 ± 0.23 upon rotenone treatment (Figure 3B). Previous reports on rotenone showed that apoptosis induction via enhancing the amount of mitochondrial reactive oxygen species production [18]. Further we checked the effects of rotenoisin B on ROS production compared with rotenone .Our data shows that rotenoisin B is able to induce ROS production from 100% ± 0.5% to 266.5% ± 1.8% after 4 h treatment, whereas rotenone from 100% ± 0.5% to 300% ± 1.8% (Figure 3B). To understand the mechanism of apoptosis caused by rotenoisin B on hepatocellular carcinoma cells compared to rotenone, we next examined the changes in pAKT signal pathway and mitogen activated protein kinases (MAPKs) involved in mediating its apoptotic action. Total cellular proteins were extracted from cells after treatment with 10 μM rotenone and rotenoisin B for a 48 h time period, and lysates were immunoblotted with a various primary antibodies. AKT phosphorylation was inhibited, resulting in down-regulation of anti-apoptotic Bcl-2 levels, pBAD as well as the up-regulation of pro-apoptotic Bax levels. The expression level of cleaved PARP and the concentration of cytochrome c in the cytoplasm increased. These results demonstrated that rotenoisin B similar to rotenone induced apoptosis in Huh-7 human hepatic cancer cells via the mitochondrial apoptosis pathway, involving the release of cytochrome c, activation of Bax, inhibition of Bcl-2, and activation of poly ADP-ribose polymerase (PARP) (Figure 3C). As showed in Figure 3D, rotenoisin B and rotenone substantially induced the activation of ERK and slightly up regulated the phosphorylation of p38 MAPK compared to control in the hepatic cancer cells. However, the molecules associated with cell proliferation and apoptosis were not changed in normal cells after treatment of 50 μM rotenoisin B (Figure 3E).

### 2.3. Rotenoisin B Effect on G_2_/M Cell Cycle Arrest

**Figure 4:**
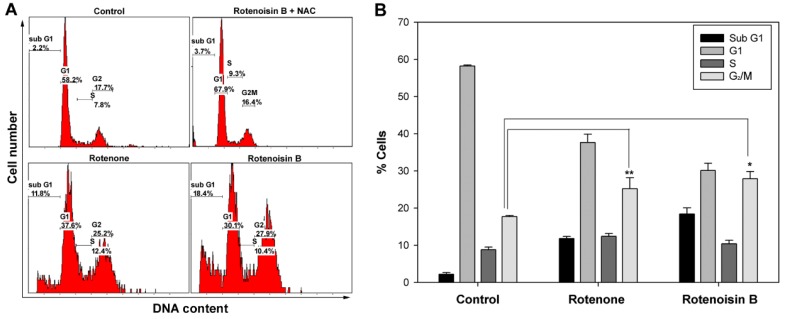
(**A**) Flow cytometry analysis of cell cycle distribution of rotenone and rotenoisin B in Huh-7 hepatic cancer cells; and (**B**) The data are the mean ± SD of the three different experiments (* *p* < 0.05, ** *p* < 0.01).

Several studies have reported rotenone induced G_2_/M cell cycle arrest and apoptosis in a human B cell lymphoma cell line, PW [19] and HepG2 cell line [20]. Our result showed rotenone and rotenoisin B both induced a significant G_2_/M cell cycle arrest and increase of sub G1. As Figure 4 showed, rotenoisin B treatment led to increase of sub G1 fraction from 2.2% ± 0.4% to 18.4% ± 1.6% and G_2_/M arrest from 7.8% ± 0.3% to 27.9% ± 1.92%. Whereas upon rotenone treatment it resulted in control increase of G_2_/M. 2.2% ± 0.4% to 11.2% ± 0.56% and an obvious G_2_/M arrest from 7.8% ± 0.3% to 25.2% ± 2.9%. In order to confirm that growth arrest was due to increased ROS by rotenone and rotenoisin B, *N*-acetyl-l-cysteine (NAC), a known antioxidant, was added, showing growth arrest was diminished (Figure 4A). These results suggested that growth arrest might be due to increased ROS by rotenone and rotenoisin B.

## 3. Materials and Methods

### 3.1. Reagent and Chemicals

Rotenone, propidium iodide (PI), Thiazolyl blue tetrazolium bromide (MTT), annexin V-FITC, protease inhibitor cocktail, and dimethyl sulfoxide (DMSO) were purchased from Sigma (Sigma Co., St. Louis, MO, USA). Antibodies for p-AKT (Ser 473), AKT, p-BAD (Ser 136), cytochrome c, poly ADP-ribose polymerase (PARP), BAX, p-ERK, ERK, p-P38, P38 and GAPDH, as well as a horseradish peroxidase (HRP)-conjugated secondary antibody were obtained from Cell Signaling Technology (Cell Signaling Tech., Beverly, MA, USA). All other chemicals used in this study were obtained from Sigma (Sigma Co., St. Louis, MO, USA).

### 3.2. Sample Preparation and Various Treatments of Stresses

Using gamma-rays, rotenone in methanol solution was directly irradiated and the converted products were monitored by HPLC, Agilent Technologies 1100 series system (Agilent Technologies, Palo Alto, CA, USA) and further column chromatographic purification led to the isolation of the new rotenone derivative, rotenoisin B [13].

### 3.3. Cell Culture and Viability Assay

Hepatic cancer cell line HepG2 and Huh-7, and Detroit 551 (ATCC CCL-110) cell lines were purchased from American Type Culture Collection (Rockville, MD, USA). Hepatic cancer cell lines were cultured with Dulbecco’s modified Eagle’s medium (DMEM) and Detroit 551 cell line was cultured with Eagle’s minimum essential medium (EMEM) supplemented with penicillin (100 units/mL), streptomycin (100 µg/mL), and 10% fetal bovine serum (FBS), and maintained in an incubator with a humidified atmosphere of 95% air and 5% CO_2_ at 37 °C. The hepatic cancer cells (1 × 10^3^) were seeded in 96-well flat-bottom plates and subsequently cells were treated with 0.1, 1, 5, 10, 20 and 40 µM or indicated concentrations rotenone, rotenoisin B and control DMSO (0.1%) respectively for 48 h. MTT at a concentration of 5 mg/mL in PBS was added to each well and incubated for another 1 h further 100 µL acidified isopropanol was added to each well. Absorbance at a wavelength of 570 nm was measured using a microplate reader (Tecan, Switzerland).

### 3.4. Evaluation of Apoptosis by Annexin-V FITC/Propidium Iodide

Apoptosis was detected by annexinV-FITC/PI double staining. Huh-7 cells were seeded in 6-well plate for 24 h. Cells were then treated with 10 µM rotenone, rotenoisin B and control DMSO (0.1%) for 48 h, respectively. After treatment, the cells were trypsinized, washed twice with PBS. Cells were resuspended in 100 µL of binding buffer (10 mM 4-(2-hydroxyethyl)-1-piperazineethanesulfonic acid (HEPES), pH 7.4, 140 mM NaCl, and 2.5 mM CaCl_2_), and incubated with annexin-V FITC for 10 min further with PI for 15 min in the dark. Annexin-V FITC and PI fluorescence were monitored using an FC500 flow cytometer (Beckman-Coulter, Fullerton, CA, USA). Ten thousand events were collected per sample. Data was analyzed using CXP analysis software (Beckman-Coulter, Fullerton, CA, USA).

### 3.5. Mitochondrial Membrane Potential (Ψ_m_) Detection by Spectroscopy

Mitochondrial membrane potential changes were assessed with JC-1 dye. In brief, cells were cultured in 6-well plate for 24 h for attachment. Further cells incubated with 10 µM rotenone and rotenoisin B for 4 h. The cells were then washed with PBS and incubated with 2.5 µg/mL JC-1 dye in PBS at 37 °C in the dark for 30 min. Cells were washed with cold PBS and visualized cells inverted florescence microscope (Olympus IX71, Olympus Corporation, Hiroyuki Sasa, Japan).

The fluorescence of JC-1 monomer alone read at excitation/emission of 485/530 nm whereas J-aggregate read at 535/590 nm using a micro plate reader (Tecan, Mannedorf, Switzerland).

### 3.6. Intracellular ROS Detection by H2DCFDA

Intracellular ROS was detected using fluorescent probe dye, 2′,7′-dichlorodihydrofluorescein diacetate (H2DCFDA). In brief, Huh-7 cells in 6-well plate were incubated with 10 µM rotenone and rotenoisin B and DMSO control for 4 h. After treatment of rotenoisin B, 3 mM *N*-acetyl-l-cysteine (NAC) was added. Cells were then trypsinized and washed in PBS and incubated with 20 µM H2DCFDA at 37 °C for 30 min in the dark. Further cells were washed with PBS and DCF fluorescence was detected using flow cytometry FC500 (Beckman-Coulter, Fullerton, CA, USA). ROS were expressed as mean fluorescence intensity (MFI), which was CXP analysis software version 2.2 (Beckman-Coulter, Fullerton, CA, USA).

### 3.7. Immuno Blot Experiment

After treatment with10 µM rotenone, rotenoisin B and control DMSO (0.1%), Huh-7 cells were scraped and lysed with radioimmunoprecipitation assay (RIPA) buffer with protease inhibitor. In order to detect cytosolic cytochrome c, cytoplasmic extraction reagent was used according to the manufacturer’s manual (Thermo Scientific Inc., Waltham, MA, USA). Protein concentrations were estimated using the colorimetric bicinchoninic acid (BCA) assay as per manufactures instructions. Gel electrophoresis was done using 12% SDS-PAGE (sodium dodecyl sulfate-polyacrylamide gel electrophoresis) gels loaded with equal sample protein amounts in each well. Gels were transferred to polyvinyl difluoride (PVDF) membrane. After blocking with 1× Tris-buffered saline containing 0.1% Tween 20 and 5% nonfat milk at room temperature for 1 h, incubated with various primary antibodies at 1:1000 dilutions for overnight, followed by goat anti-rabbit HRP-conjugate secondary antibody at 1:2000 dilutions, detection of protein expressions performed using the enhanced chemiluminescence (ECL) plus chemiluminescence kit (GE Healthcare, Buckinghamshire, UK).

### 3.8. Cell Cycle Analysis by Flow Cytometry

Cells were treated with 10 µM rotenone, rotenoisin B, and control DMSO (0.1%) for 48 h and then cells harvested by trypsinization and washed with PBS. Cells (1 × 10^6^) were resuspended in 70% Ethanol (kept at −20 °C) 5 mL drop wise manner then cells were stored at 4 °C for overnight for fixation. Cells were washed with PBS and suspended staining solution containing 0.5% Triton X-100, 10 µg/mL RNAse-A, 20 µg/mL PI, 1 mM EDTA and incubated at room temperature for 20 to 30 min in dark. Cells were then analyzed for DNA content profile by flow cytometry FC500 flow cytometer (Beckman-Coulter, Fullerton, CA, USA) from 10,000 events per sample. Data from flow cytometry was analyzed using CXP analysis software version 2.2 (Beckman-Coulter, Fullerton, CA, USA).

### 3.9. Statistical Analysis

Statistical analysis was performed using Student's unpaired t test with Sigma Plot 10.0 software. Data in figures given as mean ± SD from three replicates at least and a value of * *p* < 0.05, and ** *p* < 0.01 was considered statistically significant.

## 4. Conclusions

This study demonstrated for the first time that gamma irradiated rotenone compound, rotenoisin B, can act as a potent inhibitor for the proliferation of hepatic cancer cells. Induction of apoptosis by rotenoisin B is confirmed via the loss of Ψ_m_, release of cytochrome c, down-regulation of antiapoptotic Bcl-2 levels as well as the up-regulation of proapoptotic Bax levels and the increased level of cleaved PARP. Collectively, these results suggest that the rotenoisin B might be potentially used as an anticancer agent through the mitochondrial apoptosis and inhibition of the Akt pathway against human hepatic cancer. Here, in our strategy, radiolytic transformation of rotenone by gamma-irradiation was used to modify the structure and decrease the toxicity of the parent compound rotenone. More systematic structural modifications together with gamma-irradiation will be performed in the future to further clarify these interesting findings in order to develop even more promising anti-cancer candidates. The radiolytic transformation of high toxic compounds by gamma-irradiation may be a good strategy for modifying the structure and decreasing the toxicity of the parent compound.
